# Integrated UPLC, bioinformatics, and in vitro analyses reveal Yiqihuoxue decoction (GSC) alleviates vascular aging by promoting autophagy

**DOI:** 10.1038/s41598-026-44263-4

**Published:** 2026-03-13

**Authors:** Yiqing Liu, Yunlu Liu, Chengkui Xiu, Meiyu Cui, Yinan Liu, Xue Wang, Yanhong Hu, Qiang Wang, Yan Lei, Jing Yang

**Affiliations:** 1 Experimental Research Center, China Academy of Chinese Medical Sciences, Beijing, 100700 China; 2 National Resource Center for Chinese Materia Medica, China Academy of Chinese Medical Sciences, Beijing, 100700 China; 3 Wangjing Hospital, China Academy of Chinese Medical Sciences, Beijing, 100102 China

**Keywords:** Yiqihuoxue decoction (GSC), Vascular aging, UPLC-LTQ-Orbitrap-MS, Network pharmacology, SIRT1-autophagy, PI3K/AKT, Cell biology, Autophagy, Senescence, Molecular biology, Bioinformatics, Computational biology and bioinformatics

## Abstract

Vascular aging constitutes a predominant risk factor for cardiovascular pathologies. Traditional Chinese Medicine (TCM) employs various formulations to mitigate age-related vascular dysfunction, among which Yiqihuoxue decoction (GSC) is clinically utilized for managing cardiovascular conditions in elderly patients. Our prior work demonstrated GSC’ s capacity to delay vascular aging in mice models and attenuate senescence in vascular endothelial cells, though its mechanistic basis remained unresolved. To address this, we conducted multi investigation combining Ultra-Performance Liquid Chromatography (UPLC), network pharmacology, and molecular dynamics (MD) simulations. UPLC identified 130 bioactive compounds in GSC, while integrative analyses (mass spectrometry, literature mining, and target prediction) revealed 792 putative targets. Intersection with 2,539 vascular aging-associated targets yielded 422 shared candidates, suggesting GSC’ s polypharmacological potential. Gene Ontology (GO) and Kyoto Encyclopedia of Genes and Genomes (KEGG) enrichment analyses highlighted autophagy-related pathways, notably PI3K/Akt and SIRT1 signaling. Cellular validation experiments in senescent human umbilical vein endothelial cells (HUVECs) demonstrated that GSC restored cell morphology and reduced SA-β-gal activity (*p* < 0.05). GSC alleviated G0/G1 phase cell cycle arrest, restored mitochondrial membrane potential, and suppressed ROS accumulation. Autophagy profiling indicated that GSC promoted autophagic flux, as evidenced by increased LC3B puncta formation in immunofluorescence assays and autophagosome accumulation observed via transmission electron microscopy. Mechanistically, GSC exerted anti-senescence effects via coordinated regulation of the SIRT1-autophagy axis and PI3K/AKT pathway inhibition. Western blotting confirmed dose-dependent upregulation of SIRT1 and downregulation of p-PI3K/p-AKT (*p* < 0.05), consistent with network pharmacology predictions. This study established GSC as a multi-component, multi-target intervention against vascular aging, with autophagy modulation serving as a central mechanism. These findings will provide a theoretical foundation for developing GSC-based therapies targeting age-related cardiovascular diseases.

**Supplementary Information:**

The online version contains supplementary material available at 10.1038/s41598-026-44263-4.

## Introduction

The World Health Organization forecasts that by 2050, individuals aged 60 and older will constitute 38% of the global population, exceeding the number of adolescents aged 10–24^1^. This demographic transition underscores the urgent need to address age-related pathologies and their comorbidities. Cardiovascular diseases, the predominant cause of mortality in the elderly, exhibit a pronounced age-dependent increase in incidence^2^. Vascular aging, a critical contributor to cardiovascular disease, is characterized by progressive structural and functional deterioration of blood vessels^3^. These age-associated changes correlate with elevated risks of hypertension, Alzheimer’ s disease, stroke, and other conditions, adversely affecting healthspan in aging populations worldwide^3^. Key molecular drivers of vascular aging include oxidative stress, mitochondrial dysfunction, chronic low-grade inflammation, genomic instability, cellular senescence, epigenetic dysregulation, disrupted proteostasis, nutrient sensing deregulation, and stem cell dysfunction^4,5^. Despite the imperative to mitigate vascular aging and its clinical consequences, no targeted therapies currently exist, underscoring the need for mechanistic and therapeutic investigations.

Yiqihuoxue decoction (GSC), a traditional herbal formulation comprising *Panax ginseng* C. A. Mey., *Panax notoginseng* (Burkill) F. H. Chen ex C. H. Chow, and *Ligusticum sinense ‘Chuanxiong’* is clinically used to manage cardiovascular symptoms in elderly patients. Previous studies from our group revealed that GSC attenuates diabetes-induced endothelial senescence via AMPK-dependent mitophagy activation^6^ and ameliorates β-galactose-induced vascular aging in mice by inhibiting endothelial progenitor cell senescence^7^. However, the molecular targets and mechanisms underlying GSC’ s anti-aging effects remain incompletely characterized, in part due to the unresolved complexity of its bioactive constituents.

The multi-component nature of GSC poses significant challenges for comprehensive chemical profiling^8^. Ultra performance liquid chromatography (UPLC) has emerged as a robust analytical platform for resolving complex mixtures, including TCMs^9^. Modern drug discovery increasingly recognizes that bioactive compounds often act on multiple targets rather than single proteins^10^, a paradigm advanced by network pharmacology. This discipline enables systematic prediction of active ingredient clusters, their molecular targets, and therapeutic networks, offering a powerful framework for studying multi-target interventions like GSC^11^.

In this research, we combined UPLC-LTQ-Orbitrap-MS and UPLC-Q-TOF/MS^E^ to characterize GSC’ s chemical constituents. Bioinformatics analyses were employed to map its potential anti-aging mechanisms, with subsequent in vitro verification comfirming critical roles for SIRT1-autophagy and PI3K/AKT pathways modulation. These findings provide a mechanistic foundation for developing GSC as a therapeutic strategy against vascular aging.

## Materials and methods

### Chemicals and reagents

GSC compounds were prepared through ethanolic extraction of three medicinal herbs: *Panax ginseng* C. A. Mey.(Jilin, China), *Panax notoginseng* (Burkill) F. H. Chen ex C. H. Chow (Yunnan, China), and *Ligusticum sinense ‘Chuanxiong’* (Sichuan, China), yielding a concentration of 3.164 g raw drug/g extract (Institute of Chinese Academy of Traditional Chinese Medicine). Resveratrol (cat. no 085K1584, Sigma-Aldrich, Shanghai, China) served as the positive control^12^.

Human umbilical vein endothelial cells (HUVECs, cat. no 8000), endothelial cell culture medium (cat. no 1001), fetal bovine serum (cat. no 0025), endothelial growth factor (cat. no 1052), penicillin/streptomycin solution (cat. no 0503), pancreatic enzyme digestion solution (cat. no 0103), and cell cryopreservation solution (cat. no 0133) were purchased from ScienCell (California, USA). Cell Aging β-Galactosidase Detection Kit (cat. no GMS10012.1) was acquired from Shanghai Jiemei Gene Pharmaceutical Technology Co., Ltd (Shanghai, China). Dansylcadaverine (Monodansyl cadaverine, MDC, cat. no 30432), chloroquine (cat. no c6628), EX527 (SIRT1 inhibitor, cat. no E7034) were purchased from Sigma-Aldrich (Shanghai, China). LY294002 (PI3K inhibitor, cat. no HY10108) was purchased from MedChemExpress (New Jersey, USA). PI3K antibody (cat. no ab191606), AKT antibody (cat. no ab8805), P16 antibody (cat. no ab211542), SIRT1 antibody (cat. no ab110304), p62 antibody (cat. no ab56416), LC3B antibody (cat. no ab192890), MnSOD antibody (cat. no ab13533) and p-p66 antibody (cat. no ab53518) were sourced from Abcam (Cambridge, UK).

### Identification of GSC compounds

GSC (0.2500 g) were accurately weighed into a 250 ml volumetric flask and dissolved in 70% methanol, followed by sonication for 30 min. The solution was then centrifuged at 5000 r/min for 10 min, and the supernatant was collected as the sample solution.

Analyses were conducted using a Waters ACQUITY UPLC I-CLASS Liquid Chromatography system equipped with an ACQUITY UPLC T3 column (2.1 × 100 mm, 1.8 μm, Waters, UK). The column was maintained at 35℃, with a flow rate of 0.30 mL/min and an injection volume of 2 µL.Differential sampling and analysis methods were employed for the various compounds in GSC. A Waters Xevo TQ-S Triple Quad Mass Spectrometer with an ESI source was used for detection in both positive and negative ion modes. Source parameters were set as follows: ion source temperature, 150℃; cone gas flow rate, 0 L/h; desolvation gas temperature, 500℃; desolvation gas flow rate, 1000 L/h; collision gas, argon.

### Predicting of the compounds and disease target

The chemical composition information of GSC was retrieved from databases (Scifinder, TCMID, TCM@Taiwan, TCMSP, CNK). Mass to charge ratio (m/z) was precisely calculated. First-level mass spectrometry data comparison was conducted to identify possible compounds. Based on it, further confirmation of the compounds was carried out through second-level mass spectrometry analysis and literature search comparison^9^.

The SMILES format for GSC compounds was downloaded from PubChem (https://pubchem.ncbi.nlm.nih.gov/). In Homo sapiens conditions, compound-related targets were obtained from the Swiss Target Prediction (http://www.swisstargetprediction.ch/) by uploading their SMILES structure. The aforementioned data were imported into Cytoscape V3.8.0, which is an open-source software used to make complicated networks visualization, to construct a compound-target interaction network. Then the top 10 important compounds were screened out by the plug-ins “CytoHubba”.

The targets related to vascular aging were obtained from the DisGeNET database (http://www.disgenet.org/), OMIM (http://www.omim.org/), GeneCards (https://www.genecards.org/), TTD (http://db.drblab.net/ttd/), DrugBank (https://www.drugbank.ca/), HAGR (https://genomics.senescence.info/). All data was collected up to December 2023, “vascular”, “aging” and “vascular aging” were the keywords and “Homo sapiens” was the organism used when searching for targets, which were standardized into official gene symbols via UniProt database (http://www.uniprot.org/). The common targets of GSC action on vascular aging were obtained by constructing Venn diagrams.

### Construction of the protein–protein interaction (PPI) network

To analyze the common targets, they were imported to the STRING database (https://string-db.org/). The organism was programmed as “Homo sapiens” and the confidence level was set to “highest confidence (0.900)”. The results were put from String into Cytoscape V3.8.0 for visualization. The top 10 targets were screened out by the plug-ins “CytoHubba”, which can be used to evaluate the importance of targets, including “Degree”, “Closeness Centrality” and “Betweenness Centrality”.

### GO and KEGG enrichment analyses

GO statistical analysis and visualization were performed using R (version 4.2.1) with *p*.adj < 0.05 & qvalue < 0.2 as critical values to obtain the biological processes involved in GSC delayed vascular aging, including biological process (BP), molecular function (MF), and cellular component (CC) ontologies.

KEGG statistical analysis and visualization were performed using R (version 4.2.1) with *p*.adj < 0.05 & qvalue < 0.2 as critical values to obtain the main signaling pathways of GSC to delay vascular aging.

### Compounds-target molecular docking

The top 10 key targets were selected as receptors based on the mining results. The crystal structure numbers of Akt1, ALB, GAPDH, TNF, SRC, VEGFA, CTNNB1, EGFR, IL1B, and STAT3 were queried in the literature. Then protein structures were downloaded using PDB database (https://www.rcsb.org/) and stored in PDB format. The top 10 active compounds were selected as ligands. The three-dimensional structures of notoginsenoside K, Ginsenoside Rk3, Senkyunolide A, Gypenoside XVII, Coniferyl ferulate, 20(S)-Ginsenoside Rh2, vanillic acid, Caffeic acid, 20(S)-Ginsenoside Rg3 and Chlorogenic acid were obtained in PubChem database (https://pubchem.ncbi.nlm.nih.gov/) and stored in SDF format. Use dockthor (https://dockthor.lncc.br/v2/) 14 for molecular docking. Choose “docking” then select the PDB file in the protein column, choose “do not use cofactors” in the cofactor column, select the SDF file in the ligand column, then pick blind docking. The docking results were visualized using Pymo12.1 software (conformation with the most negative binding energy was selected for each target) to obtain the binding patterns of the compounds and proteins, according to which the amino acid residues of the compounds binding to the protein pockets could be clearly seen.

### Cell culture and experimental design

Human umbilical vein endothelial cells (HUVECs) were maintained in complete medium (5% fetal bovine serum, endothelial growth factor, 100 U/L penicillin and 100 mg/L streptomycin) at 37 °C under 5% CO_2_. Experimental groups included: Control (5th-passage cells) Model (12th-passage cells), DMSO (12th-passage cells treated with 1 µL/mL DMSO), Resv (12th-passage cells treated with 8 µmol/L resveratrol), GSC intervention: 12th-passage cells treated with GSC at high (GSC-H, 400 mg/L), medium (GSC-M, 300 mg/L), or low (GSC-L, 200 mg/L) doses for 24 h post-attachment. Additional groups were established to probe mechanistic pathways. SIRT1 inhibition: Model+EX527, GSC-H+EX527, and RESV+EX527. Autophagy/SIRT1 co-inhibition: Model + Cho+EX527, GSC + Cho+EX527, and Resv + Cho+EX527. PI3K/AKT modulation: Model+LY294002 and GSC+LY294002. Cells were seeded in 6-well plates at 1.5 × 10⁵ cells/well. Autophagy assays employed a 2-hour starvation protocol with EBSS, optimized via pre-experiments.

### Cell senescence assessment

#### β-galactosidase staining

Post-treatment, cells were washed with DPBS and incubated with β-galactosidase cleaning solution (5 min, room temperature). After aspiration, β-galactosidase acidic solution was applied, washed off, and reapplied. Pre-warmed staining solution was added, and plates were incubated overnight at 37 °C in a CO_2_ incubator. Senescence-specific β-galactosidase-positive cells were visualized as blue under an inverted microscope, and their proportion was quantified relative to total cells.

#### Cell cycle analysis

Post-treatment cells were fixed in 70% ethanol (overnight, 4 °C), treated with RNase A (37 °C, 30 min), stained with propidium iodide (PI; 4 °C, 30 min), and analyzed by flow cytometry.

#### Mitochondrial ROS fluorescence intensity

Cells were incubated with 5 µM MitoSOX Red (37 °C, 10 min, dark), washed, and imaged via fluorescence microscopy.

#### Mitochondrial membrane potential assessment

A 1x JC-10 staining solution was prepared by diluting 50 µL of 100x JC-10 with 5 mL Buffer A. Cells were stained with 50 µL of this solution for 30 min at 37 °C in a 5% CO2 incubator, protected from light. After removal, 50 µL of Buffer B was added. Fluorescence ratios at 490 nm/525 nm (green) and 540 nm/590 nm (red) were measured in a microplate reader to determine mitochondrial membrane potential.

### Cell autophagy evaluation

#### Cellular autophagosomes by transmission electron microscopy

Cells (excluding Control) were starved in EBSS (2 h), trypsinized, pelleted, and fixed in 2% glutaraldehyde (4 °C, overnight). After osmium tetroxide post-fixation and uranyl acetate staining, samples were dehydrated, embedded, and sectioned for transmission electron microscopy imaging.

#### Autophagic vesicles by MDC staining

Cells were incubated with 50 µmol/L MDC (37 °C, 1 h), fixed with 4% paraformaldehyde (15 min), and imaged via confocal microscopy (ex/em: 380/525 nm). Cells were starved in EBSS for 2 h to induce autophagy.

#### mRFP-GFP-LC3 dual fluorescent system for autophagosome labeling

Cells were treated and co-incubated with mRFP-GFP-LC3 viral solution for 6 h, then cultured in normal medium for 24 h. Autophagy and flux were observed via confocal microscopy, utilizing distinct RFP and GFP fluorescence. Cells were starved in EBSS for 2 h to induce autophagy.

#### LC3B immunofluorescence

Fixed cells were permeabilized (0.2% Triton X-100), blocked with serum, incubated with LC3B primary antibody (1:500, 4 °C, overnight) and fluorescent secondary antibody (1:1000, 37 °C, 1 h), counterstained with DAPI, and imaged by confocal microscopy. Cells were starved in EBSS for 2 h to induce autophagy.

### Western blot analysis

Cell lysates (lysis buffer, 1 h ice incubation) were quantified via BCA assay, denatured (100 °C, 10 min), separated by SDS-PAGE (100 V stacking, 150 V resolving), and transferred to PVDF membranes. After blocking (5% skim milk, 1 h), membranes were incubated with primary antibodies (4 °C, overnight) and HRP-conjugated secondary antibodies (2 h). Bands were visualized using ECL and quantified with ImageJ.

### Statistical analysis

Experimental data were analyzed using IBM SPSS Statistics 21.0 software. For normally distributed data, one-way ANOVA was applied, with post-hoc LSD tests for significant variances identified by chi-square tests. Non-parametric comparisons were made using the Kruskal-Wallis test when data deviated from normality. Data were presented as $\bar{X}$ ± s, with significance set at *P* < 0.05. Graph Pad Prism 9.0 was utilized for graphical representation of statistical outcomes.

## Results

### Compounds identification in GSC

**Fig. 1:**
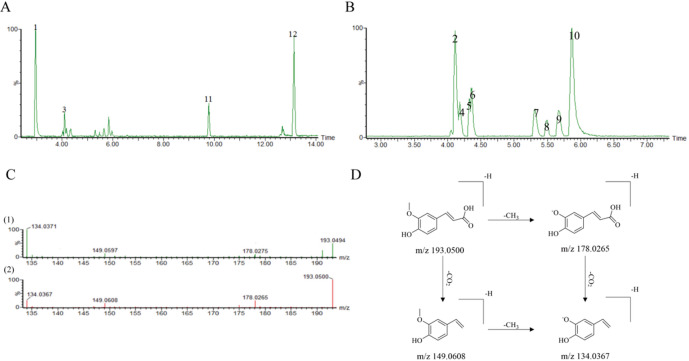
(**A**) Base peak ion current of GSC in positive ion mode. (**B**) Base peak ion current of GSC in negative ion mode. (**C**) Mass spectra of ferulic acid, (C1) Secondary mass spectra of ferulic acid, (C2) Primary mass spectra of ferulic acid. (**D**) Mass spectrometry fragmentation laws of ferulic acid.

UPLC-LTQ-Orbitrap-MS and UPLC-Q-TOF/MS^E^ identified 130 constituents in GSC, including 31 protopanaxadiol saponins, 28 protopanaxatriol saponins, 1 oleanolic acid saponins, 30 special saponins, 19 phthalide monomers, 12 phthalide Dimers, 5 acid ingredients and 3 miscellaneous components. Comparative mass spectrometry and literature reviews comfirmed 39 bioactive compounds^13–17^. Positive- and negative-ion mode profiles of GSC are showed in Table 1, while total ion chromatograms for both modes are illustrated in Fig. 1A and B. Representative fragmentation patterns are shown for ferulic acid (Fig. 1C) and its derived mass spectral cleavage pathways (Fig. 1D).

**Table 1:** Identification of 39 chemical compounds from GSC by UPLC-Q-TOF/MSE.

Num.	Identified compounds	Ion mode	Rt(min)	Formula	Measured value(m/z)	ppm
1	notoginsenoside K	-	6.12	C_48_H_82_O_18_	945.543	0.74
2	Gypenoside XVII	-	6.47	C_48_H_82_O_18_	945.5413	−1.058
3	Ginsenoside Rb1	-	5.30	C_54_H_92_O_23_	1107.5945	−0.542
4	Ginsenoside Rc	-	5.49	C_53_H_90_O_22_	1077.5861	1.485
5	Ginsenoside F2	-	8.56	C_42_H_72_O_13_	783.4885	−1.276
6	20(S)-Ginsenoside Rg3	-	8.71	C_42_H_72_O_13_	783.4892	−0.383
7	Ginsenoside Rs1	-	6.25	C_55_H_92_O_23_	1119.5801	−13.398
8	(+)-notoginsenoside R4	-	4.99	C_59_H_100_O_27_	1239.6447	5.889
9	Quinquenoside III	-	7.25	C_50_H_84_O_19_	987.5604	7.595
10	Ginsenoside Ra2	-	5.41	C_58_H_98_O_26_	1209.6274	0.496
11	notoginsenoside FP2	-	5.22	C_58_H_98_O_26_	1209.6281	1.075
12	20(S)-Ginsenoside Rh2	-	20.44	C_36_H_62_O_8_	621.4377	1.77
13	Floranotoginsenoside M	-	5.63	C_53_H_90_O_22_	1077.5859	1.299
14	Notoginsenoside R1	-	4.18	C_47_H_80_O_18_	931.5269	0.322
15	Ginsenoside Re	-	4.33	C_48_H_82_O_18_	945.5429	0.635
16	Ginsenoside Rg1	-	4.36	C_42_H_72_O_14_	799.4835	−1.126
17	20(S)-Ginsenoside Rh1	-	5.89	C_36_H_62_O_9_	637.4312	−0.628
18	Ginsenoside F3	-	5.53	C_41_H_70_O_13_	769.4736	−0.26
19	Ginsenoside Rf	-	5.33	C_42_H_72_O_14_	799.4832	−1.501
20	Ginsenoside Ro	-	5.66	C_48_H_76_O_19_	955.4907	0.419
21	Ginsenoside Rg2	-	5.77	C_42_H_72_O_13_	783.4901	0.766
22	Notoginsenoside M	-	4.10	C_48_H_82_O_19_	961.538	0.832
23	Ginsenoside Rk1	-	10.48	C_42_H_70_O_12_	765.4789	0
24	Notoginsenoside T5	-	7.53	C_41_H_68_O_12_	751.463	−0.399
25	Ginsenoside F4	-	7.50	C_42_H_70_O_12_	765.4789	0
26	Ginsenoside Rk2	+	5.50	C_36_H_60_O_7_	605.4446	4.79
27	Ginsenoside Rk3	+	7.97	C_36_H_60_O_8_	621.4367	0.161
28	Z-butylidenephthalide	-	4.43	C_12_H_12_O_2_	187.0968	0
29	Senkyunolide A	+	8.54	C_12_H_16_O_2_	193.1228	−0.518
30	Coniferyl ferulate	+	18.97	C_20_H_20_O_6_	379	0
31	Levistilide A	+	13.12	C_24_H_28_O_4_	381.2068	0.525
32	Riligustilide	+	11.64	C_24_H_28_O_4_	381.2078	3.148
33	Chlorogenic acid	-	2.37	C_16_H_18_O_9_	353.0873	−4.248
34	vanillic acid	-	3.41	C_8_H_8_O_4_	167.0357	7.783
35	Caffeic acid	-	2.83	C_9_H_8_O_4_	179.0343	−0.559
36	p-Hydroxybenzoic acid	-	4.94	C_7_H_6_O_3_	137.0223	−11.677
37	kaempferol	-	5.87	C_15_H_10_O_6_	285.0399	0
38	Tetramethylpyrazine	+	2.96	C_8_H_12_N_2_	137.1082	2.188
39	Quercitrin	+	4.10	C_21_H_20_O_11_	449.109	1.336

### Compounds-disease targets network construction

**Fig. 2:**
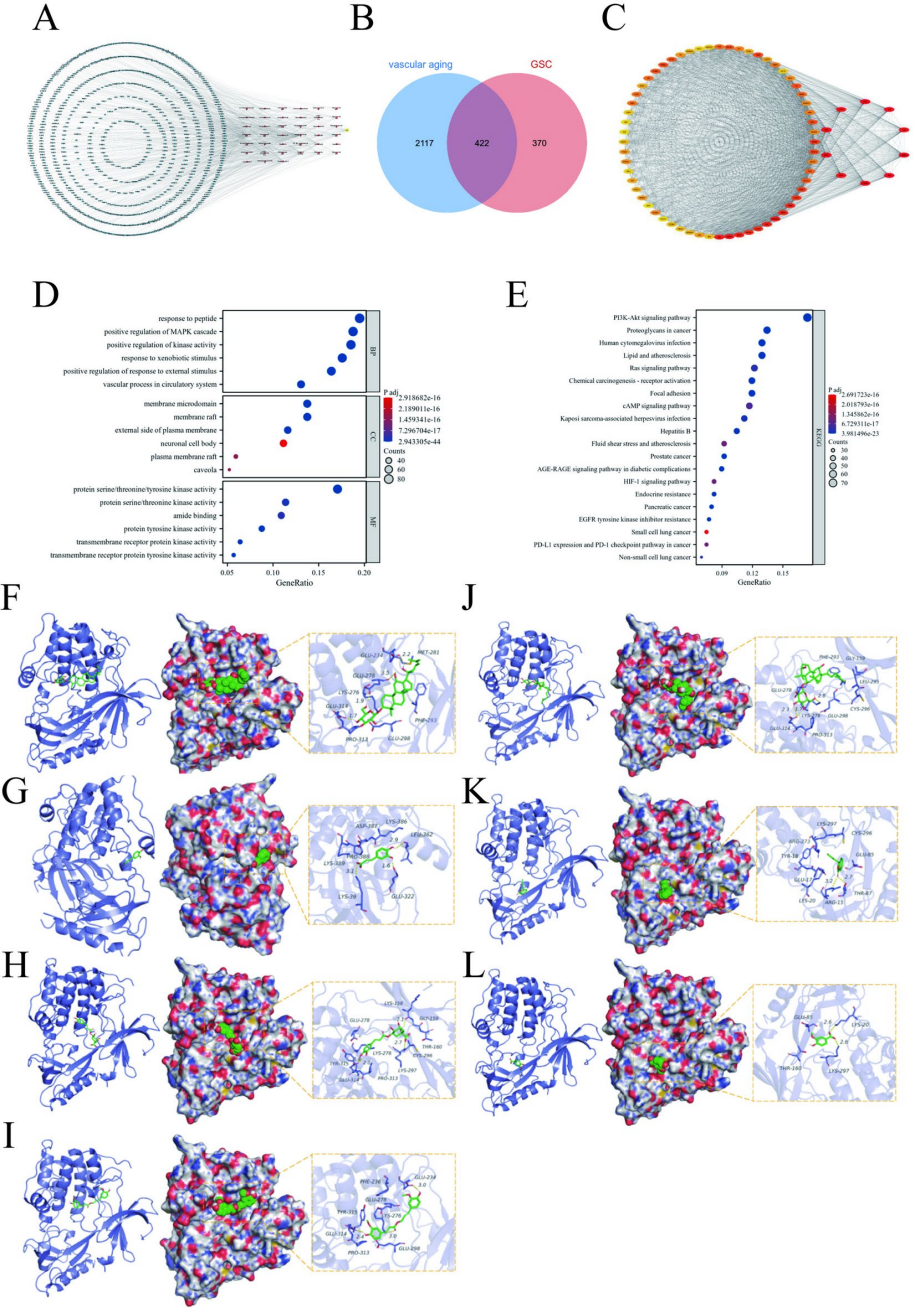
(**A**) GSC-compounds-related targets network. (**B**) 422 common targets of compounds and disease. (**C**) Core target-protein interaction network of GSC in the treatment of vascular aging. The darker the color, the more critical is the node in the network. (**D**) Bubble diagram of the GO functional enrichment. (**E**) Bubble diagram of the KEGG pathway enrichment. The KEGG pathway maps were reproduced with permission from Kanehisa Laboratories^18^. (**F**) 20(S)-Ginsenoside Rh2, (**G**) Caffeic acid, (**H**) Chlorogenic acid, (**I**) Coniferyl ferulate, (**J**) Ginsenoside Rk3, (**K**) Senkyunolide A, (**L**) vanillic acid.

SMILES format structures of the 39 bioactive compounds were retrieved from PubChem, and 940 putative targets were predicted using Swiss Target Prediction. After deduplication, 792 compound-related targets remained (Fig. 2A). The top-ranked compounds included notoginsenoside K, Ginsenoside Rk3, Senkyunolide A, Gypenoside XVII, Coniferyl ferulate, 20(S)-Ginsenoside Rh2, vanillic acid, Caffeic acid, 20(S)-Ginsenoside Rg3 and Chlorogenic acid. Vascular aging-related targets (2539) were collated from DisGeNET, OMIM, GeneCards, TTD, DrugBank and HAGR. Intersection analysis revealed 422 shared targets between GSC compounds and vascular aging, suggesting GSC’ s multi-target therapeutic potential (Fig. 2B).

### Protein-protein interaction (PPI) network analysis

The 422 shared targets were analyzed using the STRING database to construct a PPI network. Node color intensity correlated with interaction degree, highlighting 70 core targets (Fig. 2C). The top 10 hub targets were AKT1 (degree = 244), ALB (degree = 237), GAPDH (degree = 231), TNF (degree = 225), SRC (degree = 198), VEGFA (degree = 198), CTNNB1 (degree = 196), EGFR (degree = 193), IL1B (degree = 176), and STAT3 (degree = 169) (Table 2).

**Table 2:** The 10 core targets of GSC in vascular aging treatment.

NO.	Name	Degree	BetweennessCentrality	ClosenessCentrality
1	AKT1	244	0.064649067	0.69628433
2	ALB	237	0.079791248	0.688498403
3	GAPDH	231	0.048559084	0.681962025
4	TNF	225	0.045672165	0.672386895
5	SRC	198	0.03871255	0.643283582
6	VEGFA	198	0.022797949	0.645209581
7	CTNNB1	196	0.026973603	0.643283582
8	EGFR	193	0.025300364	0.639465875
9	IL1B	176	0.020854088	0.624637681
10	STAT3	169	0.012743671	0.613960114

### GO and KEGG enrichment analyses

The 422 common targets were imported to R (version 4.2.1) for GO functional enrichment and KEGG pathway enrichment. GO analysis identified 3438 biological process (BPs), 342 cellular compounds (CCs), and 353 molecular functions (MFs) (*p*.adj < 0.05, qvalue < 0.2). Top 6 enriched terms were selected for visualization (Fig. 2D). Based on the enrichment analysis of BPs, the targets were associated mainly with response to peptide, positive regulation of MAPK cascade, positive regulation of kinase activity, response to xenobiotic stimulus and positive regulation of response to external stimulus. CCs mainly included membrane microdomain, membrane raft, external side of plasma membrane, neuronal cell body and plasma membrane raft. MFs included protein serine, threonine, tyrosine kinase activity, amide binding and protein tyrosine kinase activity.

**Fig. 3:**
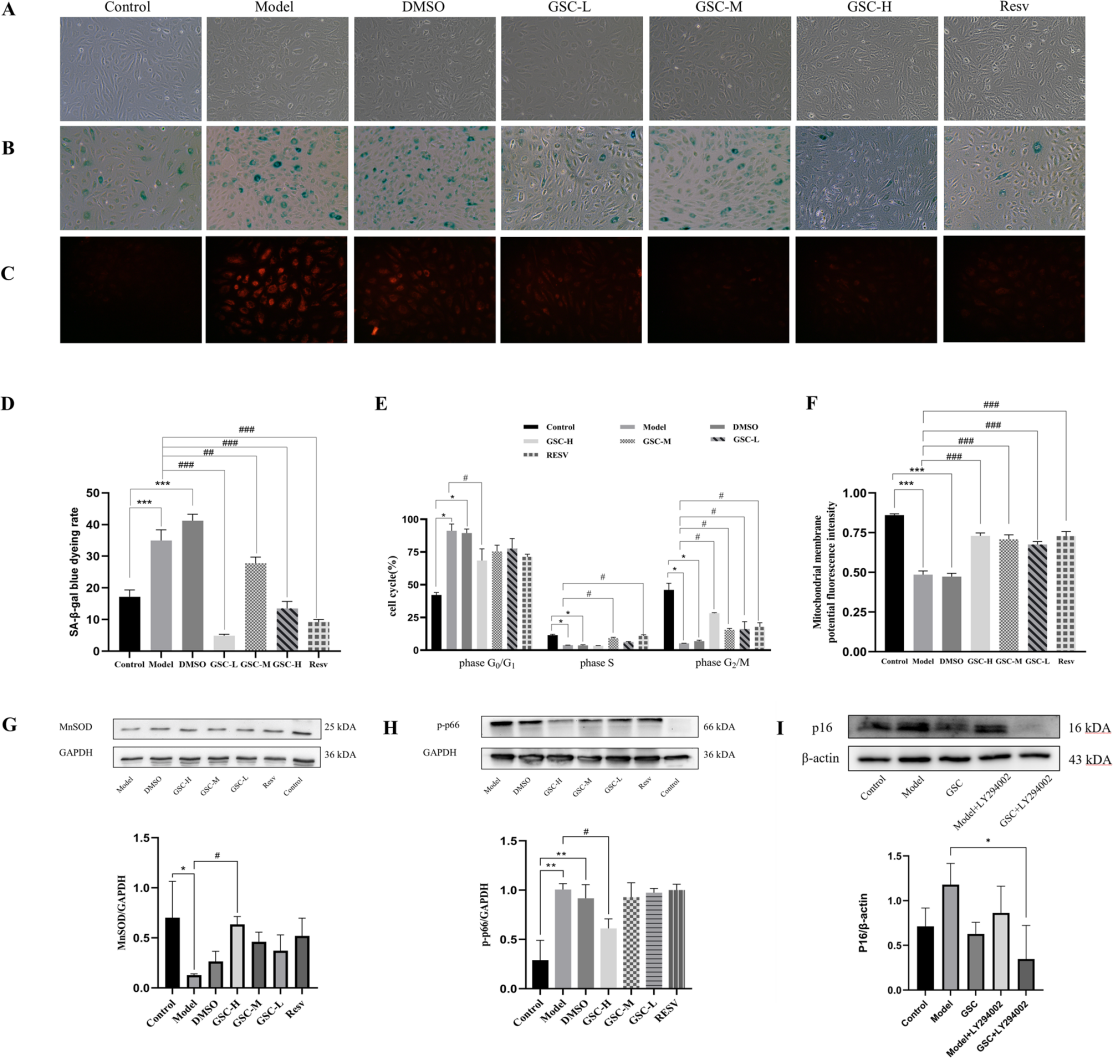
GSC ameliorated cellular senescence via oxidative stress and cell cycle regulation. (**A**) Morphological changes of cells in each group under a light microscopy. (**B**) SA-β-gal staining visualized under light microscopy. (**C**) Quantification of mitochondrial ROS fluorescence intensity. (**D**) SA-β-gal-positive cell percentage calculated from blue-stained cells. (**E**) Flow cytometry analysis of Cell cycle distribution. (**F**) Detection of mitochondrial membrane potential. (**G**) Western blot analysis of MnSOD normalized to GAPDH. (**H**) Western blot analysis p-P66 normalized to GAPDH. (**I**) Western blot analysis of p16 protein levels normalized to β-actin. LY294002 was used at a concentration of 2 µmol/L simultaneously. Data are presented as means ± SEM (*n* ≥ 3). ^*^p < 0.01 vs. control group, ^#^*p* < 0.05 vs. model group.

KEGG pathway analysis (192 pathways, *p*.adj < 0.05, qvalue < 0.2) prioritized 20 pathways (Fig. 3E.), including PI3K/Akt signaling pathway, Proteoglycans in cancer, Human cytomegalovirus infection, Lipid and atherosclerosis and Ras signaling pathway (Table 3). Bubble plots (Fig. 2D and E) visualize term ratios (x-axis), pathway categories (y-axis), target counts (bubble size), and enrichment significance (color gradient: bluer= stronger enrichment).

**Table 3:** The top 20 pathway enrichment of GSC in vascular aging treatment.

ID	Pathway item	Count	*p* value
hsa04151	PI3K-Akt signaling pathway	76	6.54E-25
hsa05205	Proteoglycans in cancer	54	1.35425E-25
hsa05163	Human cytomegalovirus infection	52	8.3183E-22
hsa05417	Lipid and atherosclerosis	52	8.76764E-23
hsa04014	Ras signaling pathway	49	1.46688E-18
hsa05207	Chemical carcinogenesis - receptor activation	48	8.91515E-20
hsa04510	Focal adhesion	48	7.96929E-21
hsa04024	cAMP signaling pathway	47	3.36525E-18
hsa05167	Kaposi sarcoma-associated herpesvirus infection	45	4.95194E-19
hsa05161	Hepatitis B	42	9.04693E-20
hsa05418	Fluid shear stress and atherosclerosis	37	6.0378E-18
hsa05215	Prostate cancer	37	4.62509E-24
hsa04933	AGE-RAGE signaling pathway in diabetic complications	36	2.00922E-22
hsa04066	HIF-1 signaling pathway	33	5.52782E-18
hsa01522	Endocrine resistance	33	1.35786E-19
hsa05212	Pancreatic cancer	32	1.5751E-22
hsa01521	EGFR tyrosine kinase inhibitor resistance	31	9.33484E-21
hsa05222	Small cell lung cancer	30	1.8311E-17
hsa05235	PD-L1 expression and PD-1 checkpoint pathway in cancer	30	6.32023E-18
hsa05223	Non-small cell lung cancer	28	9.92529E-19

### Compounds-target molecular docking validation

Molecular docking utilized binding energy ≤ − 5.0 kJ/mol as the stability threshold. Despite unresolved crystal structures for GAPDH, IL1B, notoginsenoside K, Gypenoside XVII, and 20 (S)-Ginsenoside Rg3, 7 core compounds exhibited stable binding (energy < − 5.0 kJ/mol) with 8 key targets. For example, caffeic acid formed hydrogen bonds with AKT1 residues (LYS-386, LEU-362, GLU-322) and van der Waals interactions with adjacent residues (ASP-387, PRO-388, LYS-389), stabilizing its position within the protein cavity (Table 4; docking modes in Fig. 2F and L).

**Table 4:** The binding energies of 7 core compounds and 8 core targets.

Compound	Akt1	ALB	TNF	SRC	VEGFA	CTNNB1	EGFR	STAT3
20(S)-Ginsenoside Rh2	−8.4	−7.8	−8.2	−9.2	−7.7	−7.3	−8.2	−7.7
Caffeic acid	−7.0	−6.3	−6.1	−6.4	−6.4	−5.9	−6.5	−6.3
Chlorogenic acid	−6.4	−7.2	−7.4	−6.9	−6.4	−6.5	−6.7	−7.1
Coniferyl ferulate	−7.4	−7.2	−7.4	−7.9	−7.8	−7.4	−7.5	−8.6
Ginsenoside Rk3	−7.9	−7.2	−8.5	−8.6	−8.2	−8.2	−8.6	−8.2
Senkyunolide A	−8.1	−7.9	−7.0	−7.4	−6.9	−6.9	−7.2	−7.6
vanillic acid	−6.7	−6.5	−6.5	−7.3	−6.4	−6.2	−6.9	−6.3

### GSC attenuated cellular senescence in HUVECs

#### GSC improved morphology and reduces SA-β-gal activity

To evaluate GSC’ s anti-senescence effects, we analyzed morphological changes and SA-β-gal activity in aging HUVECs. Compared to Control group, Model and DMSO groups exhibited slowed proliferation, cytoplasmic vacuolization, multinucleation, reduced adhesion, and partial cell detachment. GSC treatment reduced cytoplasmic vacuoles and granules while restoring cell adhesion (Fig. 3A). SA-β-gal staining intensity, significantly elevated in Model and DMSO groups, was dose-dependently suppressed by GSC (Fig. 3B and D), confirming its senescence-delaying capacity.

#### GSC reversed cell cycle arrest, restores mitochondrial membrane potential, and attenuates ROS accumulation

Flow cytometry revealed G0/G1 phase arrest in Model group cells, with reduced S and G2/M phase populations. GSC-H restored cell cycle progression, decreasing G0/G1 phase cells while increasing S and G2/M phase populations (Fig. 3E).

Mitochondrial dysfunction in Model groups was evidenced by mitochondrial membrane potential loss (Fig. 3F) and elevated mitochondrial ROS fluorescence (bright red signals, Fig. 3C). GSC-H, GSC-M, and Resv groups exhibited partial mitochondrial membrane potential restoration and intermediate ROS levels between Control and Model groups, indicating mitochondrial functional recovery.

#### GSC modulated oxidative stress in senescent cells

As the primary mitochondrial ROS scavenger, MnSOD protein levels reflect oxidative stress severity. Model group cells showed significant MnSOD downregulation (*p* < 0.05 vs. control). All GSC doses and Resv elevated MnSOD expression, with GSC-H achieving statistical significance (*p* < 0.05), demonstrating GSC ’ s capacity to mitigate mitochondrial oxidative stress (Fig. 3G). Oxidative stress triggers p-p66 translocation to mitochondria, amplifying ROS production. Model groups exhibited marked p-p66 upregulation (*p* < 0.05 vs. control), which was attenuated by GSC-H (Fig. 3H). Senescence-associated p16 overexpression in model groups was suppressed post-GSC intervention, suggesting cell cycle reentry (Fig. 3I).

### GSC modulated cellular autophagy in HUVECs

#### GSC promotes autophagosome formation observed by transmission electron microscopy

**Fig. 4:**
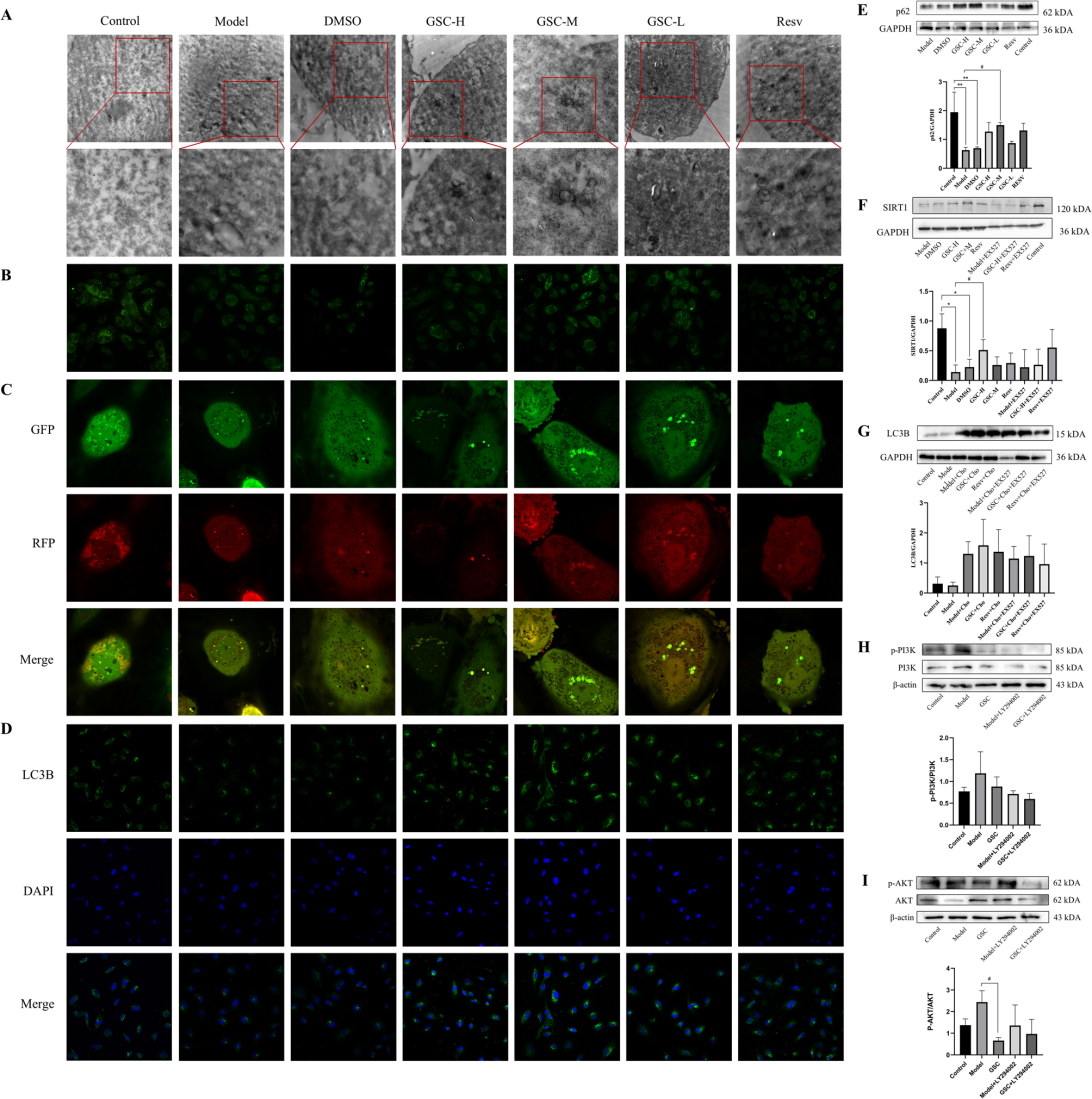
GSC modulated SIRT1-autophagy and PI3K/Akt in senescent cells. (**A**) Transmission electron microscope images of autophagosomes in each group (×20,000 magnification for main panel; ×50,000 magnification for inset). (**B**) MDC staining detected autophagic vesicle changes. (**C**) mRFP-GFP-LC3 dual-fluorescence system tracking of autophagic flux. (**D**) Laser confocal microscopy quantification of LC3B puncta fluorescence intensity. (A-D) The cells in each group were given EBSS for 2 h for starvation autophagy except for the Control group in electron microscope experiments. (**E**) Western blot analysis of p62 expression normalized to GAPDH. (**F**) Western blot analysis of SIRT1 protein levels normalized to GAPDH. EX527 (10 µmol/L) was included in combination experiments. (**G**) Western blot analysis of LC3B normalized to GAPDH. Chloroquine (50 µmol/L) was co-administered to block autophagosome-lysosome fusion. (**H**) Western blot analysis of p-PI3K/PI3K normalized to β-actin. (**I**) Western blot analysis of p-AKT/AKT normalized to β-actin. LY294002 (25 µmol/L) was used to inhibit PI3K signaling. Data are presented as mean ± SEM (*n* ≥ 3). *p < 0.05 vs. control group; ^#^*p* < 0.05 vs. model group. Autophagy induction was confirmed by EBSS starvation for 2 h.

Electron microscopy revealed distinct autophagic structures across groups. Control group cells displayed intact nuclei, uniform chromatin, and sparse lysosomes. In contrast, Model and DMSO groups exhibited membrane damage, chromatin disorganization, and abundant autophagosomes/autolysosomes with double-membrane structures. GSC-treated groups showed further increases in autophagosomes and secondary lysosomes, while Resv displayed the most pronounced autophagy activation (Fig. 4A).

#### MDC staining indicates GSC enhances autophagic vesicle accumulation

MDC fluorescence intensity, reflecting autophagosome formation, was reduced in Model group compared to Control. Treatment with GSC and Resv increased the MDC signal intensity (Fig. 4B).

#### mRFP-GFP-LC3 assay confirms GSC restores autophagic flux

We utilized the mRFP-GFP-LC3 tandem fluorescent probe to assess autophagic flux. In this system, yellow puncta (GFP+/RFP+) represent autophagosomes that have not fused with lysosomes, while red-only puncta (GFP-/RFP+) indicate autolysosomes where the acid-sensitive GFP signal has been quenched. In the Model group, we observed a mix of both yellow and red puncta. In contrast, cells treated with GSC or Resveratrol displayed a notable increase in the number of red-only puncta relative to yellow puncta (Fig. 4C). This shift in the puncta color profile indicates that GSC not only induces autophagy but, more importantly, promotes the efficient completion of autophagic flux by enhancing the fusion of autophagosomes with lysosomes to form autolysosomes.

#### GSC increases LC3B puncta formation

Chloroquine-mediated inhibition of autophagosome-lysosome fusion elevated LC3B fluorescence. GSC and Resv groups exhibited higher LC3B signal than Model groups (*p* < 0.05), confirming autophagy induction (Fig. 4D).

#### GSC regulates the expression of key autophagy-related proteins

Elevated p62 protein levels are usually considered as a sign of inhibited autophagic activity. Compared to Control group, the p62 protein level in Model group was significantly downregulated (*p* < 0.05), while GSC-H, GSC-M and Resv groups were increased (*p <* 0.05). Contrary to our previous expectation, we speculate this may be due to context-dependent p62-autophagy interplay (Fig. 4E). Model groups exhibited SIRT1 suppression (*p* < 0.05), reversed by GSC-H (*p* < 0.05). EX527 abrogated GSC’ s effects, confirming SIRT1-dependent autophagy regulation (Fig. 4F).

Chloroquine acts as an inhibitor of autophagy and autophagic flow by hindering the fusion of autophagosomes and lysosomes. When chloroquine was not added to Control and Model groups, LC3B protein loss was shown. However, LC3B protein content was elevated in GSC + Cho group compared to Model + Cho group. The protein content was lower in GSC + Cho+EX527 group compared to GSC + Cho group, indicating EX527 further inhibited the autophagy level of senescent cells by inhibiting the SIRT1 pathway. LC3B level decreased in the GSC + Cho group after administration of EX527, further implicating GSC’ s effect on autophagy through the SIRT1 pathway (Fig. 4G).

### GSC modulated PI3K-AKT pathway in HUVECs

Model groups exhibited elevated p-PI3K/PI3K and p-AKT/AKT ratios, attenuated by GSC (*p* < 0.05). LY294002 synergized with GSC to reduce p16 expression, suggesting GSC mitigates senescence via PI3K/AKT suppression, potentially counteracting chronic inflammation during replicative aging (Fig. 4H and I).

## Discussion

Aging, an irreversible biological process, amplifies susceptibility to chronic diseases such as hypertension, diabetes, and atherosclerosis. Vascular aging—marked by structural and functional degeneration of endothelial cells—serves as a critical precursor to cardiovascular pathologies^19^. Traditional Chinese Medicine (TCM) posits that aging stems from deficiencies in qi (vital energy) and blood circulation. GSC, a TCM formulation containing Panax ginseng, Panax notoginseng, and Ligusticum chuanxiong, has demonstrated anti-aging potential by attenuating aortic thickening in D-galactose-induced aged mice^20^. However, its bioactive components and mechanistic underpinnings remained unexplored.

Using UPLC and mass spectrometry, we identified 39 bioactive compounds from 130 GSC constituents, including notoginsenoside K, ginsenoside Rk3, and caffeic acid. Network pharmacology predicted 792 targets, with molecular docking confirming stable interactions (binding energy < − 5.0 kJ/mol) between 7 key compounds (e.g., 20(S)-ginsenoside Rg3) and 8 hub targets (AKT1, TNF, IL1B). Protein-protein interaction (PPI) analysis prioritized AKT1, GAPDH, and VEGFA as central mediators of GSC’ s effects. Enrichment analyses revealed two pathways: (1) the cAMP-SIRT1 signaling pathway. It regulates cell growth, differentiation, and gene transcription^21^. Researchers have found cAMP could response to diminished smooth muscle relaxation in blood vessels from older animals^22^ and resveratrol attenuates endothelial inflammation by inducing autophagy. Autophagy was mediated through the activation of the cAMP-PRKA-AMPK-SIRT1 signaling pathway^23^. (2) the PI3K/Akt signaling pathway. It involves in cell cycle, cell proliferation, survival signal and activates multiple signaling pathways. In the previous studies, significant activation of PI3K/Akt signaling was observed in replicative senescence of human vascular smooth muscle cells^24^, in age-induced cerebral vascularity male rats, and in senescent vascular endothelial cell^25^.

In replicative senescent HUVECs, GSC restored cell morphology, reduced SA-β-gal activity, and reversed G0/G1 phase arrest. It elevated mitochondrial membrane potential and MnSOD expression, mitigating ROS accumulation—a hallmark of oxidative stress-driven senescence^26–29^. Paradoxically, GSC increased p62 levels despite autophagy activation, likely reflecting temporal delays in autophagic flux degradation rather than suppression^30–32^. This aligns with evidence that p62-LC3II complex dynamics are context-dependent, particularly under oxidative stress^31^^32^,.

Critically, GSC upregulated SIRT1, counteracting its age-related decline. EX527 (SIRT1 inhibitor) abolished this effect, confirming SIRT1’ s role in autophagy-mediated senescence delay. These findings mirror resveratrol’ s SIRT1-dependent lifespan extension in nematodes^33^, underscoring conserved mechanisms. Concurrently, GSC suppressed p-P66—a senescence-associated protein that amplifies mitochondrial ROS under oxidative stress^34–36^—highlighting its dual role in mitigating oxidative and inflammatory axes.

Unexpectedly, while PI3K/AKT activation typically suppresses senescence, its hyperactivation in aged cells exacerbated replication stress. GSC reduced p-PI3K/PI3K and p-AKT/AKT ratios, synergizing with LY294002 (PI3K inhibitor) to downregulate p16. This paradox aligns with evidence that PI3K/AKT exerts context-dependent roles: its inhibition attenuates telomere damage^37^, yet chronic inflammation—a hallmark of aging—may aberrantly activate the pathway^38,39^. Our data suggest GSC normalizes PI3K/AKT dysregulation, potentially decoupling its pro-survival and pro-aging effects.

While this study elucidates GSC’ s anti-aging mechanisms through a multi-faceted approach, several limitations warrant attention and pave the way for future research. Firstly, while our pharmacological data using chloroquine strongly support the role of enhanced autophagic flux, genetic validation using ATG5 or ATG7 knockdown models was not performed. It is important to note that the chloroquine-based autophagic flux assay employed here is a well-established method to demonstrate the dependency of a biological process on functional autophagy. Our significant findings in Fig. 4G therefore provide robust evidence for the engagement of this pathway. Future studies employing such genetic approaches are essential to conclusively establish the causal necessity of autophagy in GSC’ s mechanism. Secondly, although EX527 inhibition confirmed the molecular dependence on SIRT1 for protein upregulation, a comprehensive analysis of its effects on a broader range of functional senescence phenotypes under SIRT1 inhibition will be valuable. Furthermore, the present findings are primarily based on a replicative senescence model. Future studies should seek to validate the efficacy of GSC in additional models of vascular aging, such as stress-induced senescence (e.g., with doxorubicin or H_2_O_2_), and should include more thorough verification of endothelial cell identity in high-passage cultures. Addressing these points in subsequent investigations will further solidify the translational potential of GSC as a therapeutic strategy against vascular aging.

## Supplementary Information

Below is the link to the electronic supplementary material.

Supplementary Material 1

## Data Availability

The datasets used and/or analysed during the current study available from the corresponding author on reasonable request.
