# Supplementary material for: Integrated UPLC, bioinformatics, and in vitro analyses reveal Yiqihuoxue decoction (GSC) alleviates vascular aging by promoting autophagy
Source: Sci Rep. 2026 Mar 13;16:13338. doi: 10.1038/s41598-026-44263-4 (PMC13106785; doi:10.1038/s41598-026-44263-4)
Supplement: Supplementary file 1 — Supplementary Material 1 [file 41598_2026_44263_MOESM1_ESM.docx]

**Title:** Integrated UPLC, bioinformatics, and *in vitro* analyses reveal Yiqihuoxue decoction (GSC) alleviates vascular aging by promoting autophagy

**Contents:** This document contains the original images of gels and blots presented in the main manuscript, in accordance with the journal's digital image and integrity policies.

**Supplementary Figure.**

1. Original blots for Fig. 3.


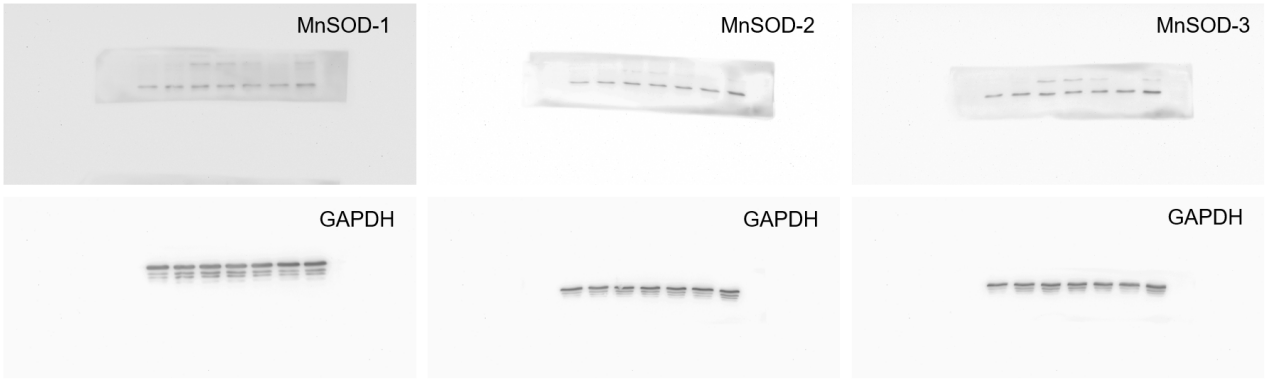


Fig. 3G


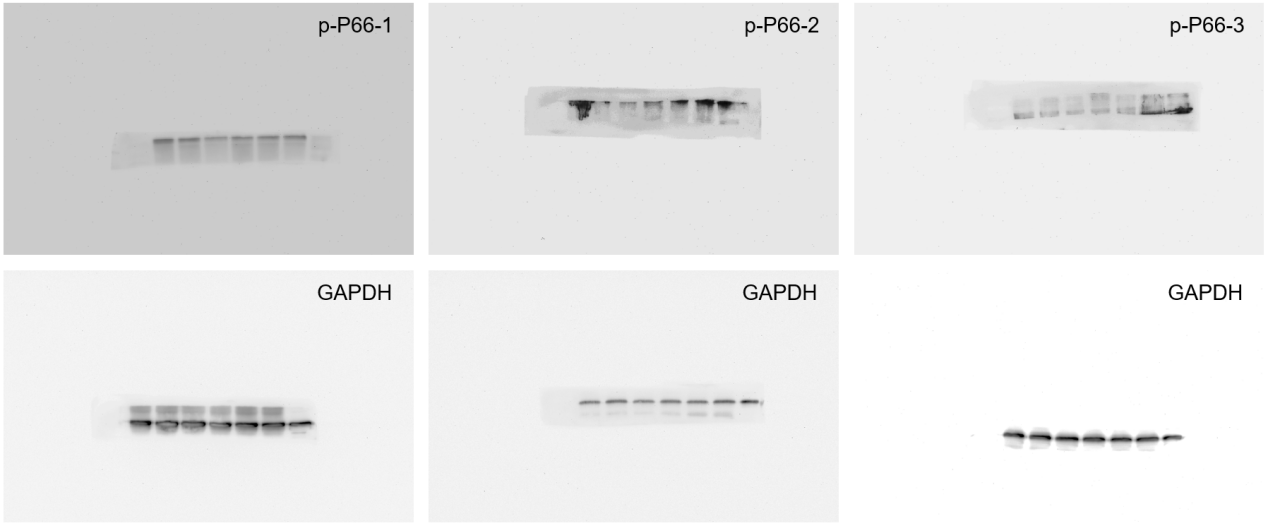


Fig. 3H


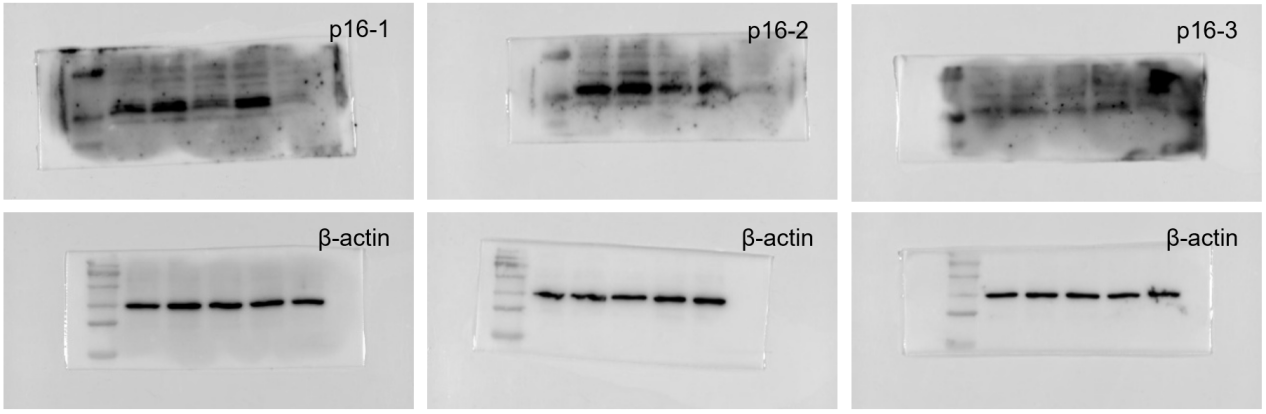


Fig. 3I

2. Original blots for Fig. 4.


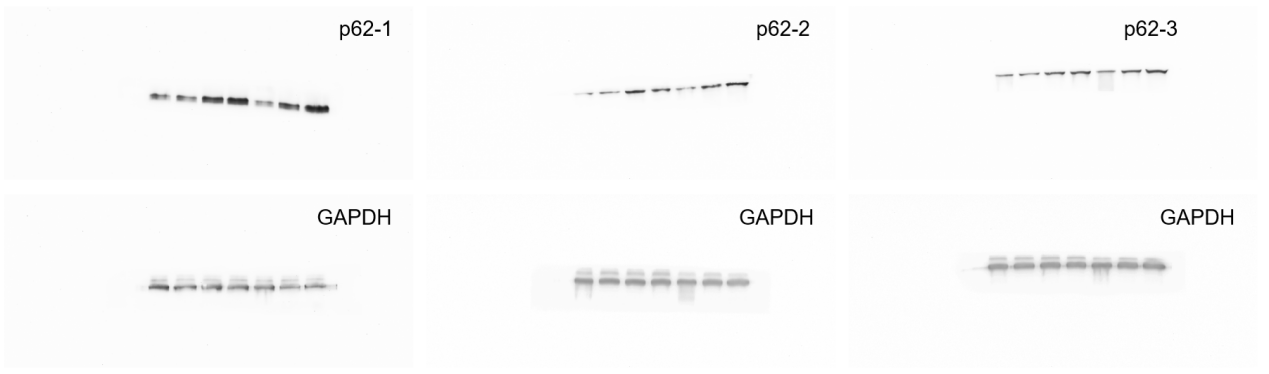


Fig. 4E


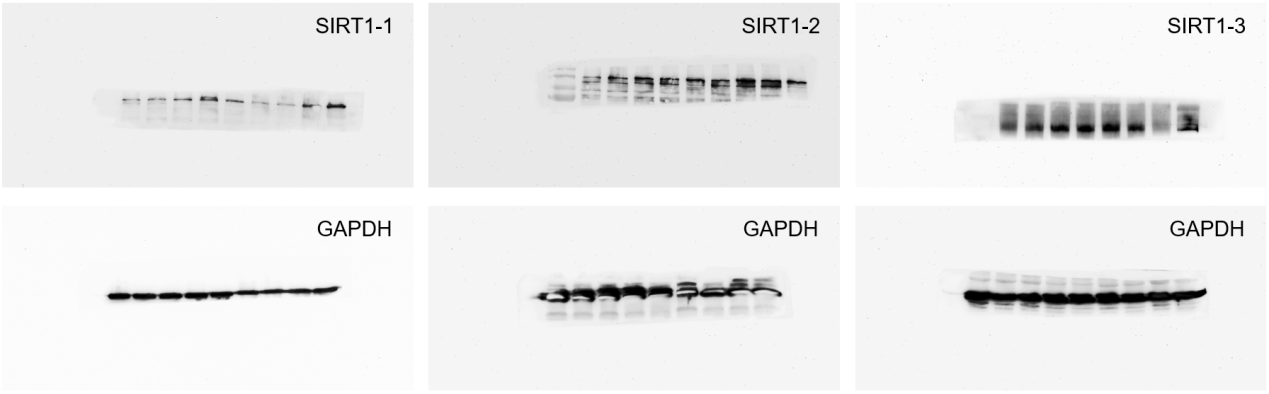


Fig. 4F


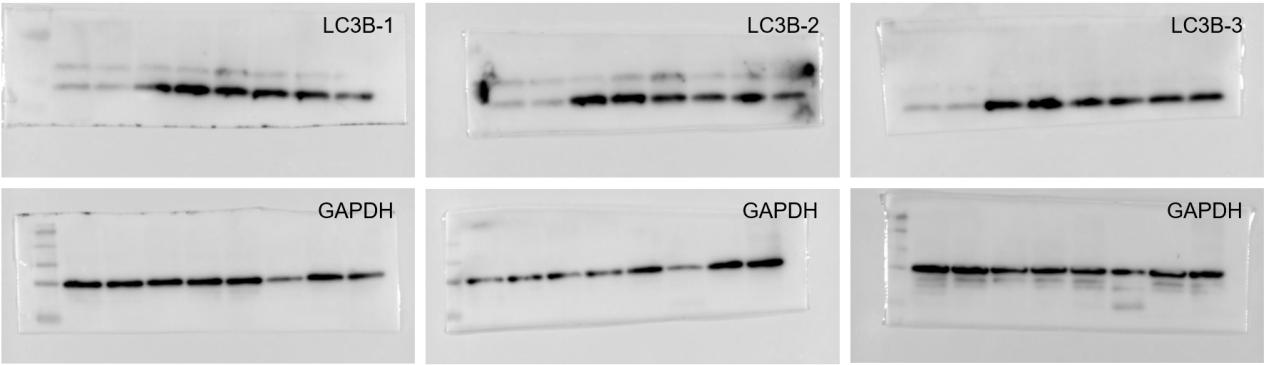


Fig. 4G


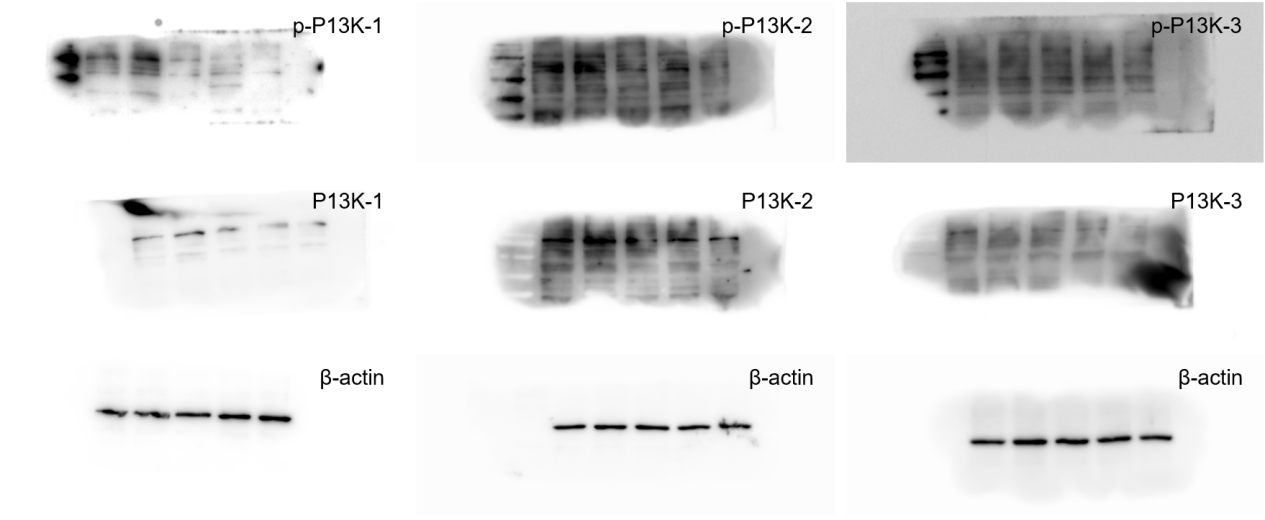


Fig. 4H


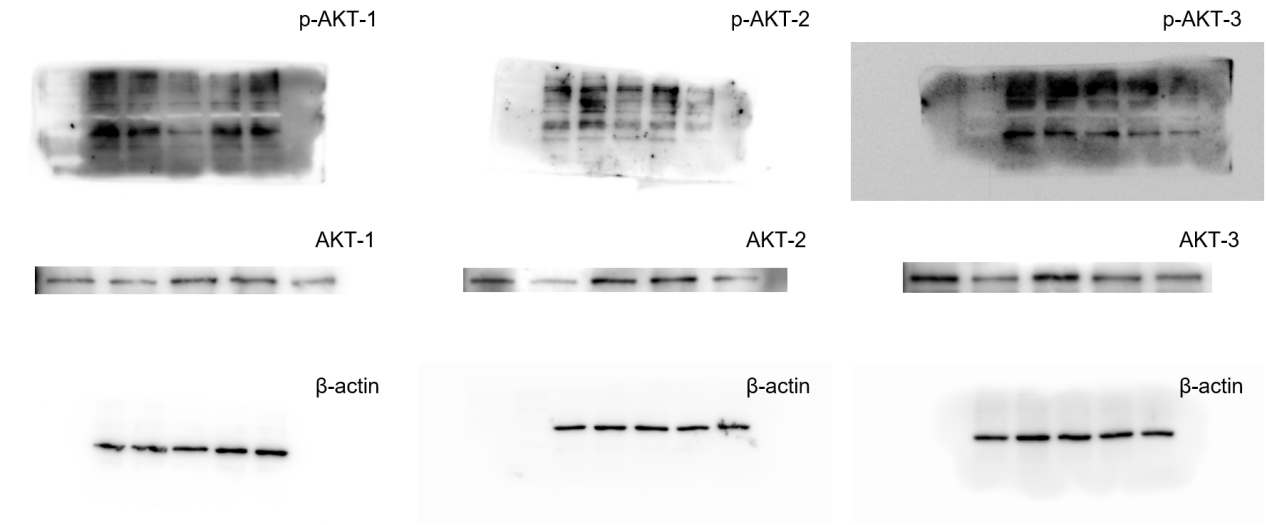


Fig. 4I
